# Detection of embedded dynamics in the Györgyi-Field model

**DOI:** 10.1038/s41598-020-77874-6

**Published:** 2020-12-03

**Authors:** Judita Buchlovská Nagyová, Branislav Jansík, Marek Lampart

**Affiliations:** 1 IT4Innovations, VSB - Technical University of Ostrava, 708 33 Ostrava, Czech Republic; 2 Department of Applied Mathematics, VSB - Technical University of Ostrava, 708 33 Ostrava, Czech Republic

**Keywords:** Chemistry, Mathematics and computing

## Abstract

The main aim of this paper is to detect embedded dynamics of the Györgyi-Field model of the Belousov–Zhabotinsky chemical reaction. The corresponding three-variable model given as a set of nonlinear ordinary differential equations depends on one parameter, the flow rate. As certain values of this parameter can give rise to chaos, an analysis was performed in order to identify different dynamics regimes. Dynamical properties were qualified and quantified using classical and also new techniques; namely, phase portraits, bifurcation diagrams, the Fourier spectra analysis, the 0–1 test for chaos, approximate entropy, and the maximal Lyapunov exponent. The correlation between approximate entropy and the 0–1 test for chaos was observed and described in detail. The main discovery was that the three-stage system of nested sub-intervals of flow rates showed the same pattern in the 0–1 test for chaos and approximate entropy at every level. The investigation leads to the open problem of whether the set of flow rate parameters has Cantor-like structure.

## Introduction

The Belousov–Zhabotinsky chemical reaction (BZ reaction), originally discovered in the 1950s by Belousov^1^, is an example of an oscillating chemical reaction which can be maintained far from equilibrium by an internal source of energy^2^ resulting in a nonlinear chemical oscillator exhibiting different dynamical regimes. Later on, the chemical mechanism of the reaction was described in^3^, and is commonly called the FKN mechanism.

There are many mathematical models representing different aspects of the BZ reaction. For example, the Brusselator, Oregonator, and Györgyi-Field are three mathematical models for a type of autocatalytic reaction, like the BZ reaction.

The Oregonator model is the result of a quantitative kinetic analysis of oscillations in the BZ reaction by Field and Noyes^4^ and is a simplified version of the mechanism developed by Field, Körös and Noyes (FKN mechanism)^1^.

The Brusselator model, a theoretical model for a type of autocatalytic reaction, was proposed by I. Prigogine and his collaborators^5^.

Finally, the Györgyi-Field model (GF model), describes a reaction taking place in a continuous-flow stirred-tank reactor (CSTR)^6^, with a relatively simple mathematical model (see also^7,8^). This model, for a specific choice of parameters, exhibits chaos (see e.g.^9^ and references therein, or the main results of this paper), contrary to the Oregonator, which has no chaotic solutions^10^ describing the oscillatory behaviour and pattern formation in the BZ reaction. The GF model will be taken into consideration for further research in this paper.

In recent decades, the BZ reaction has been studied extensively by physical chemists for its kinetic behaviour^9,11,12^, and by mathematicians for the dynamics and patterns of the solutions of the associated mathematical model^10,13–15^.

More specifically, the research was done from the theory of dynamical systems point of view. The transitions from steady state to quasi-periodic and bursting oscillations, and further on to regular relaxation oscillation via a complicated sequence of alternating periodic and chaotic regimes were achieved through by simulations in^16^. The results of computer experiments on information processing in a hexagonal array of vesicles filled with BZ solution in a sub-excitable mode were introduced by^17^. The discretized version of BZ reaction models was also researched. E.g. in^18^, the dynamics of the local map are discussed, and the set of trajectories that escape to infinity as well as the set of bounded trajectories are analyzed, i.e., the Julia set of the system. The evidence of chaos was also demonstrated experimentally by dozens works e.g.^19–21^. The emergence of chaotic oscillations in closed unstirred batch reactors has been attributed to the coupling among chemical kinetics and transport phenomena, following a Ruelle–Takens–Newhouse like scenario. In particular, transport phenomena due to concentration and density gradient were found to play a fundamental role^22–26^.

Despite a large number of results in the given area, it is instructive to apply new methods to the given BZ reaction model and to obtain new very interesting results that better characterize the trajectory behaviour depending on the choice of state parameters showing properties of the parameter space.

This work focuses on the characterization of the dynamical properties of the GF model^6^, depending on the flow rate, denoted by $k_f$, and detection of its embedded dynamics. The qualitative and quantitative characterization of the dynamics regimes is mainly done using the maximal Lyapunov exponent, the 0–1 test for chaos, and approximate entropy. Recall that the last two aforementioned tools were applied in^27^ to the two-dimensional coupled map lattice model of the Lagrangian type, which is a discrete version of the BZ reaction. These tools were applied to the voluminous simulation data generated by the Salomon supercomputer at IT4Innovations National Supercomputing Center located in Ostrava, the Czech Republic.

The paper is organized as follows: in “The Györgyi-Field reaction model” section the model is introduced, followed by its mathematical model in “Mathematical model” section. The main results obtained by phase portraits, the Fourier spectra analysis, the maximal Lyapunov exponent, the approximate entropy, and the 0–1 for chaos are contained in “Main results” section. Finally, the outcomes are summarized in “Discussion” section.

## The Györgyi-Field reaction model

The GF model of the BZ reaction develops a description of the reaction in terms of a set of differential equations containing only three variables. In common with chemical experiments, the GF model shows both regular, intermittent and chaotic behavior. While remaining close to a real chemical system, it is sufficiently simple to allow detailed mathematical analysis^6^. The mechanism of the reaction is defined by the set of the following equations (1):

1a $$\begin{aligned} Y+X+H \rightarrow 2V \end{aligned}$$

1b $$\begin{aligned} Y+A+2H \rightarrow V+H \end{aligned}$$

1c $$\begin{aligned} 2X \rightarrow V \end{aligned}$$

1d $$\begin{aligned} 0.5 X+A+H \rightarrow X+Z \end{aligned}$$

1e $$\begin{aligned} X+Z \rightarrow 0.5 X \end{aligned}$$

1f $$\begin{aligned} V+Z \rightarrow Y \end{aligned}$$

1g $$\begin{aligned} Z+M \rightarrow \end{aligned}$$

where the corresponding chemical components are: $Y=$ Br^−^, $X=$ HBrO_2_, $Z=$ $\hbox {Ce}^{4+}$, $V=$ BrCH(COOH)_2_ or BrMA, $A=$ BrO_3_^−^, $H=$ H^+^, and $M=$ CH_2_(COOH)_2_. The concentrations of the main reactants $A$, $H$, $M$, and the total concentration of cerium ions $C$ are summarized in Table 2.

## Mathematical model

A three-variable mathematical model of the BZ reaction, presented by Györgyi and Field in^6^, describes a reaction taking place in a CSTR. The corresponding set of nonlinear ordinary differential equations contains only three variables, while still being able to accurately reproduce the behavior of the BZ reaction observed experimentally^6^, and it is based on a four-variable chemical mechanism (1), see^6^.

The mathematical model, in its dimensionless form, consists of a set of scaled differential equations:

2a $$\begin{aligned} \frac{dx}{d\tau }&=T_0 ( -k_1 HY_0 x{\tilde{y}}+k_2 A H^2 Y_0/X_0 {\tilde{y}}-2k_3 X_0 x^2+0.5 k_4 A^{0.5} H^{1.5}X_0^{-0.5}(C-Z_0 z)x^{0.5} \nonumber \\&\quad -\,0.5 k_5 Z_0 xz -k_f x) \end{aligned}$$

2b $$\begin{aligned} \frac{dz}{d\tau }=T_0 \left( k_4 A^{0.5} H^{1.5} X^{0.5} (C/Z_0 -z)x^{0.5}-k_5 X_0 xz -\alpha k_6 V_0 zv-\beta k_7 Mz - k_f z \right) \end{aligned}$$

2c $$\begin{aligned} \frac{dv}{d\tau }=T_0 \left( 2k_1 HX_0 Y_0/V_0 x {\tilde{y}}+k_2 AH^2 Y_0/V_0 {\tilde{y}}+k_3 X^2_0/V_0 x^2-\alpha k_6 Z_0 zv -k_f v \right) \end{aligned}$$

where

3 $$\begin{aligned} \tau = t/T_0, x = X/X_0, z = Z/Z_0, v = V/V_0, \end{aligned}$$

and ${\tilde{y}}= \left( \alpha k_6 Z_0 V_0 zv / \left( k_1 HX_0 x +k_2 AH^2 + k_f \right) \right) /Y_0$ while $t$ corresponds to time, $X$ to HBrO_2_, $Y$ to Br^−^, $Z$ to $\hbox {Ce}^{4+}$, and $V$ to BrMA. The rate constants and parameters used in the following computations are given in Tables 1 and 2, respectively.

**Table 1:** Rates and rate constants of the GF model chemical scheme.

Reaction equation	Rate $r_i$	Rate constant $k_i$
(1a)	$r_1 = k_1 HYX$	$k_1 = 4.0 \times 10^6 \,\mathrm{M}^{-2}\,\mathrm{s}^{-1}$
(1b)	$r_2 = k_2 AH^2 Y$	$k_2 = 2.0 \,\mathrm{M}^{-3}\,\mathrm{s}^{-1}$
(1c)	$r_3 = k_3 X^2$	$k_3 = 3000 \,\mathrm{M}^{-1}\,\mathrm{s}^{-1}$
(1d)	$r_4 = k_4 A^{0.5}H^{1.5}(C-Z)X^{0.5}$	$k_4 = 55.2 \,\mathrm{M}^{-2.5}\,\mathrm{s}^{-1}$
(1e)	$r_5 = k_5 XZ$	$k_5 = 7000 \,\mathrm{M}^{-1}\,\mathrm{s}^{-1}$
(1f)	$r_6 = \alpha k_6 ZV$	$k_6 = 0.09 \,\mathrm{M}^{-1}\,\mathrm{s}^{-1}$
(1g)	$r_7 = \beta k_7 MZ$	$k_7 = 0.23 \,\mathrm{M}^{-1}\,\mathrm{s}^{-1}$

**Table 2:** Parameters of the investigated system (2).

List of parameters	
$$A = 0.1$$	
$$M = 0.25$$	
$$H = 0.26$$	
$$C = 0.000833$$	
$$\alpha = 666.7$$	
$$\beta = 0.3478$$	

The behavior of this system depends on the inverse residence time of the CSTR, and the flow rate, noted $k_f$ [${\rm s}^{-1}$]. As certain values of this parameter can give rise to chaos, the following analysis was performed in order to identify different dynamics.

## Main results

The system of differential equations (2) was solved numerically in Matlab^28^ using the $ode45$ solver. The simulations were done depending on the free parameter $k_f$ ranging from $3 \times 10^{-4}$ to $5 \times 10^{-4}$ with a $10^{-7}$ step. Each simulation was performed for the final time $\tau = 100$ with a time step of $10^{-4}$. To avoid system distortions, only the last 20% of simulations were used for further computations. In all cases, the initial conditions were set as

4 $$\begin{aligned} x_0 =z_0 = v_0 = 1. \end{aligned}$$

The choice of initial conditions is given by (3); the variables describe the ratio between the concentration at a certain time and the initial concentration.

As a main result, phase diagrams, amplitude frequency spectra (FFT), and Poincaré sections were done for relevant choices of the parameter $k_f$. To illustrate changes in dynamical behavior, bifurcation diagrams underlined by the approximate entropy and the 0–1 test for chaos with suitable magnifications to the parameter $k_f$ were plotted.

Consequently, and as a goal of this paper, bifurcation diagrams together with the maximal Lyapunov exponent, the approximate entropy, and the 0–1 test for chaos were computed for the nested set of parameters $k_f$. The 0–1 test for chaos splits the values of the parameter for which regular (periodic or quasi-periodic) and irregular (chaotic) trajectories appear, while the output of approximate entropy detects increasing complexity of the investigated system (2). Meanwhile, the Lyapunov exponents of a system describe the rate at which the orbits in phase space of two nearby points converge or diverge as time evolves. Obviously, a system’s dimension equals the number of the Lyapunov exponents. The system’s dynamics approaches equilibrium if all these exponents are negative; if at least one equals zero and the remaining are negative, a limit cycle situation has occurred; and finally, where any exponent is positive there is chaos^29^.

### Phase diagrams, the Fourier spectra, bifurcation diagrams, and the Lyapunov exponents

**Figure 1:**
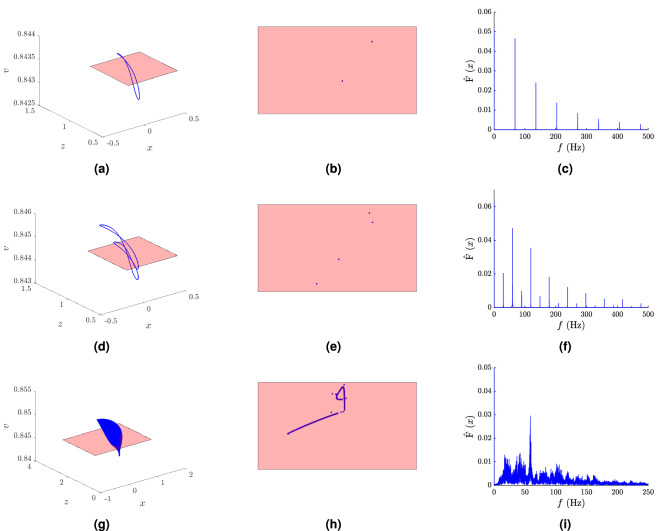
Phase portraits, Poincaré sections and Fourier spectra for different choices of the parameter $k_f$. (**a**) Regular trajectory as a trivial loop for $k_f=3 \times 10^{-4}$, (**b**) Poincaré section for $k_f=3 \times 10^{-4}$ showing 2 points of intersection, (**c**) Fourier spectra of harmonic frequencies for $k_f=3 \times 10^{-4}$; (**d**) regular trajectory showing a loop for $k_f=3.2 \times 10^{-4}$, (**e**) Poincaré section for $k_f=3.2 \times 10^{-4}$ showing 4 points of intersection, (**f**) Fourier spectra of harmonic frequencies for $k_f=3.2 \times 10^{-4}$; (**g**) chaotic trajectory for $k_f=3.5 \times 10^{-4}$, (**h**) Poincaré section for $k_f=3.5 \times 10^{-4}$ showing a band of points of intersection, (**i**) chaotic Fourier spectra for $k_f=3.5 \times 10^{-4}$.

Periodic as well as chaotic dynamics were identified in the studied model (2). For example, in Fig. 1, regular trajectory is demonstrated by the trivial loop (Fig. 1a) for $k_f=3\times 10^{-4}$, and the non-trivial loop (Fig. 1d) for $k_f=3.2\times 10^{-4}$. Figure 1g gives an example of a chaotic trajectory; $k_f=3.5\times 10^{-4}$.

The Fourier spectra were computed using the Fast Fourier transform for $k_f=3\times 10^{-4}$, $k_f=3.2\times 10^{-4}$, and $k_f=3.5\times 10^{-4}$, shown in Fig. 1c,f,i respectively. Regular behavior is observable for the first two, and chaos in the last case.

In the case of regular trajectory, the Fourier spectra in Fig. 1c,f are formed by a number of harmonic frequencies, hence the frequency of the periodic trajectory is computable. Periodic motions of trajectory are also visible as isolated points on the Poincaré sections in Fig. 1b,e.

In the case of chaos, seen in Fig. 1i, the Fourier spectra are formed by a number of harmonic components having the basic, super-harmonic, sub-harmonic, and combination frequencies on which further motions with frequencies forming the sided bands of the dominant frequencies are superposed. Their mutual ratio indicates the irregularity of the motion. The character of this chaotic motion is underlined by the band of points on the Poincaré section in Fig. 1h.

**Figure 2:**
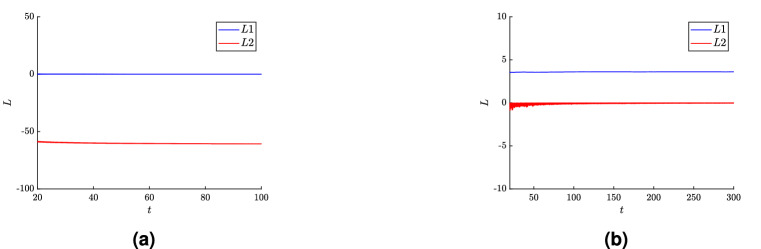
Graphs of the Lyapunov exponents for: (**a**) regular trajectory for $k_f=3.2 \times 10^{-4}$ and (**b**) chaotic trajectory for $k_f=3.5 \times 10^{-4}$. Only the largest two exponents, denoted by *L*1 and *L*2, are displayed since the third one is sufficiently negative and it has no influence on the identification of chaos.

To underline the dynamics behaviour of the selected examples shown in Fig. 1 their Lyapunov exponents are calculated and are given in Fig. 2. The maximum of these coefficients is called the maximal Lyapunov exponent and is denoted by *L*. In the case of the flow rate $k_f=3.2 \times 10^{-4}$, shown in Fig. 2a, L approaches 0, detecting a cycle, and for the flow rate $k_f=3.5 \times 10^{-4}$, shown in Fig. 2b, L is 3.604, indicating chaotic regime; in this figure only two Lyapunov exponents are displayed, the third one is omitted since it is sufficiently negative, hence, it has no influence on trajectory type.

**Figure 3:**
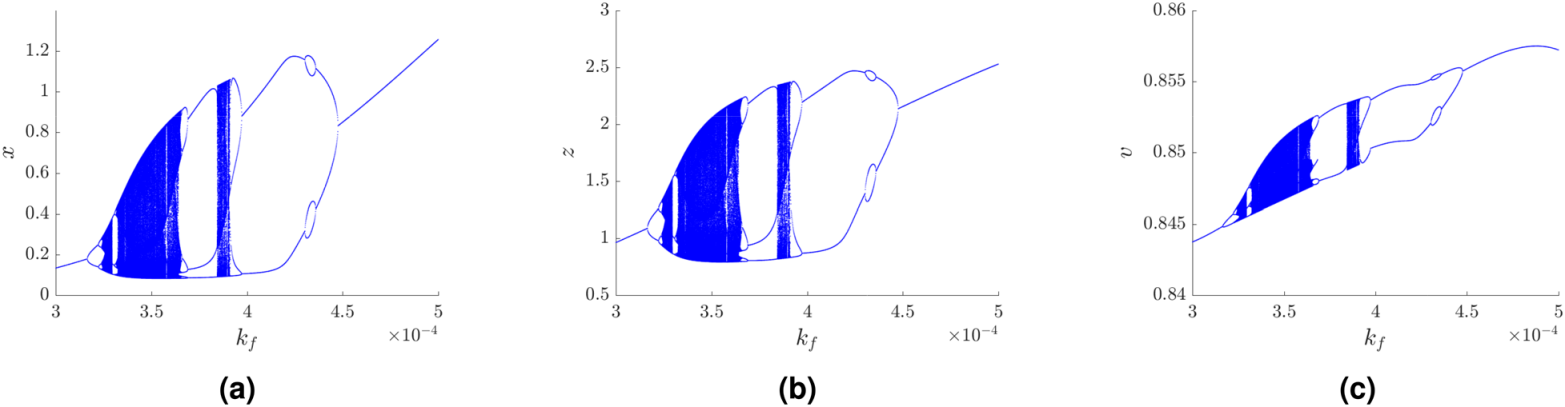
Bifurcation diagrams for the parameter $k_f \in [3 \times 10^{-4},5 \times 10^{-4}]$ for variables: (**a**) $x$, (**b**) $z$, and (**c**) $v$.

Next, the bifurcation diagram (constructed as a projection of the local maxima) of the model (2) was plotted for each variable $x$, $z$, and $v$ with respect to the free parameter $k_f \in (3 \times 10^{-4}, 5 \times 10^{-4})$ in Fig. 3. In this bifurcation diagram, so-called “period doubling” and “windows” effects are also visible. Periodic trajectory can be identified in the range of the parameter, e.g., $k_f \in (3 \times 10^{-4}, 3.24 \times 10^{-4})$ and $k_f \in (3.95 \times 10^{-4}, 5 \times 10^{-4})$. The interval in between these values is interrupted by chaotic cases around $k_f = 3.25$, and some chaotic cases for $k_f \in (3.34 \times 10^{-4}, 3.65 \times 10^{-4})$ and $k_f \in (3.85 \times 10^{-4}, 3.9 \times 10^{-4})$. As it is visible in Fig. 3 there are not only blocks of $k_f$ parameters of high system’s complexity followed by a block of parameters corresponding to cycles, but also reverse bifurcation is observable (e.g., starting at $k_f=3.6$ and ending at $k_f=3.75$). This means that the complexity decreases while system’s parameter increases.

### Approximate entropy

The approximate entropy is a technique used to quantify the amount of regularity and unpredictability of fluctuations in time-series. The main advantages are that it can be computed on short time series and it allows comparison of the differences in complexity of the same system with different parameter settings, see, e.g.,^30^. More complex notions of entropy type can be found in, e.g.,^31^. To compute the approximate entropy, two parameters must be set: embedding dimension *m* and neighborhood threshold *r*. Let $s(t) \in {\mathbb {R}}$ for $t = \{1,2,\dots ,N\}$ be a time series with *N* observations. Then embedded vector *S*(*t*) at time *t*, is defined as $S(t) = [s(t), s(t+1),s(t+2),\dots ,s(t+(m-1))]$, where *t* is the observed time and *m* is the embedding dimension. The maximum distance of embedded vectors is computed as follows:

$$\begin{aligned} D(i, j) = d(S(i),S(j)) = \max _{k=0,1,\dots ,m-1}|s(i + k) - s(j + k)|, \end{aligned}$$

for $i,j = \{1,2,\dots ,N-(m-1)\}$. Compute the thresholded version of the distance with the threshold given by *r*:

$$\begin{aligned} d_r(i,j) = \left\{ \begin{array}{ll} 1, &{}\quad {}D(i,j) < r \\ 0, &{}\quad {}~otherwise, \\ \end{array}\right. \end{aligned}$$

for $$i,j \in \{1,2,\dots , N-(m-1)\}.$$

Compute $C_i^m(r)$ as a ratio between points in the neighborhood of i and the number of embedded vectors.

$$\begin{aligned} C_i^m(r) = \frac{\sum _{j=1}^{N-(m-1)} d_r(i,j)}{N-(m-1)}. \end{aligned}$$

Then compute the average of the logarithm of all the $C_i^m(r)$

$$\begin{aligned} \Phi ^m(r) = \frac{1}{N-(m-1)} \sum _{i=1}^{N-(m-1)}\ln C^m_i(r). \end{aligned}$$

Finally, approximate entropy for the finite time series with *N* data points is computed as

$$\begin{aligned} ApEn(m,r,N) = \Phi ^m(r) - \Phi ^{m+1}(r). \end{aligned}$$

For robust estimation, it was suggested by Pincus^30^ that a time series contains at least $10^3$ observations.

The approximate entropy was calculated using the *TSEntropies* package^32^ for R^33^. The computations were made for the input vector *s* given in a normalized form of all state variables:

$$\begin{aligned} s(t) = \sqrt{x^2(t)+z^2(t)+v^2(t)}, \end{aligned}$$

$k_f\in (3 \times 10^{-4},5 \times 10^{-4})$ and $r=0.1$. The results of approximate entropy for all values of the parameter $k_f$ are in Fig. 6.

### 0–1 test for chaos

The 0–1 test for chaos, invented by Gottwald and Melbourne^34^, is one of the methods for distinguishing between regular and chaotic dynamics of a deterministic system. In contrast to the other approaches, the nature of the system is irrelevant, thus the test can be applied directly to experimental data, ordinary differential equations, or partial differential equations. The results return values close to either 0 or 1, with 0 corresponding to regular dynamics and 1 to chaotic dynamics. With its easy implementation, evaluation, and wide range of application, using this tool for detecting chaos is becoming more popular in different fields^35–38^.

The 0–1 test for chaos can be computed by the following algorithm^34^.

**Figure 4:**
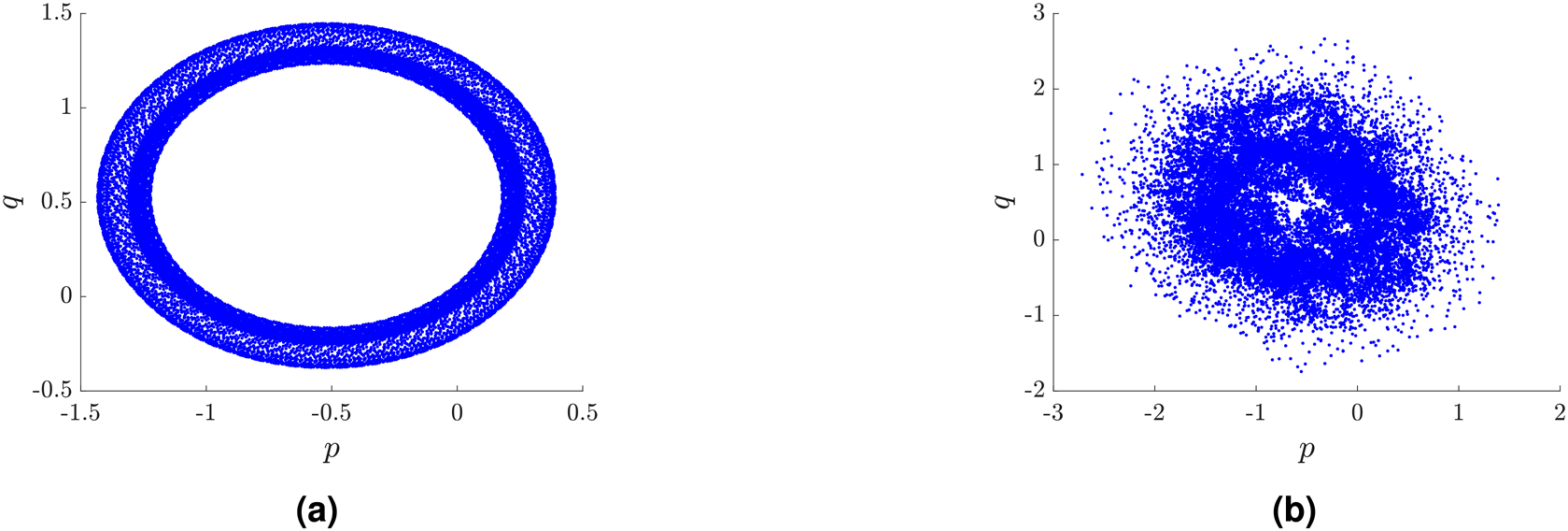
A plot of $p$ versus $q$ for $c=1.569853$: (**a**) for $k_f = 3\times 10^{-4}$ showing regular dynamics, (**b**) for $k_f = 3.5\times 10^{-4}$ showing chaotic dynamics.

Given the observation $\phi (j)$ for $j=1,2,\ldots ,N$ and a suitable choice of $c \in (0,2\pi )$, the following translation variables are computed:

$$\begin{aligned} p_c(n)& {}= \sum _{j=1}^{n}\phi (j)\cos (jc),\\ q_c(n)& {}= \sum _{j=1}^{n}\phi (j)\sin (jc) \end{aligned}$$

for $n=1,2,\ldots ,N$. The dynamics of the translation components $p_c$ and $q_c$ are shown on the $p_c$ versus $q_c$ plot. A bounded trajectory is shown in Fig. 4 (left) corresponding to regularity, for $k_f = 3\times 10^{-4}$. An unbounded trajectory is shown in Fig. 4(right) related to the chaotic case, for $k_f = 3.5\times 10^{-4}$.

The idea for the 0–1 test, first described in^34^, is that the boundedness or unboundedness of the trajectory $\{(p_{j},q_{j})_{j\in [1,N]}\}$ can be studied through the asymptotic growth rate of its time-averaged mean square displacement (MSD), which is defined as

$$\begin{aligned} M(n)=\lim _{N \rightarrow \infty }\frac{1}{N}\sum _{j=1}^{N}d(j,n)^{2} \end{aligned}$$

where

$$\begin{aligned} d(j,n)=\sqrt{(p_{j+n}-p_{j})^{2}+(q_{j+n}-q_{j})^{2}} \end{aligned}$$

is the time lapse of the duration $n$ $(n\ll N)$ starting from the position at time $j$. As shown in^34,39^, it is important to use values of $n$ small enough compared to $N$, noted $n_{cut}, (n \le n_{cut})$. A subset of time lags ${n_{cut} \in [1,N/10]}$ is advised for the computation of each $K_{c}$.

For bounded trajectories and regular dynamics, $M(n)$ is a bounded function in time, whereas unbounded trajectories, meaning chaotic dynamics, are described by $M(n)$ growing linearly with time. Thus the asymptotic growth rate of the MSD must be calculated, which correlates with the unboundedness of the trajectory.

As proposed in^34^, the modified MSD is calculated as

$$\begin{aligned} D(n)=M(n)-E(\phi )^{2}\frac{1-\cos (nc)}{1-\cos c} \end{aligned}$$

The output of the 0–1 test for chaos is computed by the correlation method as

$$\begin{aligned} K_{c}={{\,\mathrm{corr}\,}}(\xi , \Delta ) \in [-1, 1] \end{aligned}$$

for the vectors $\xi = (1, 2, \ldots , n_{cut})$ and $\Delta = (D_c(1), D_c(2), \ldots , D_c(n_{cut}))$.

The final result of the test is

$$\begin{aligned} K= {{\,\mathrm{median}\,}}(K_{c}). \end{aligned}$$

The position of the studied system (2) at any moment of time is determined by displacements *x*, *z*, and *v*, which are used for defining vector *s*:

$$\begin{aligned} s(t) = \sqrt{x^2(t)+z^2(t)+v^2(t)}. \end{aligned}$$

**Figure 5:**
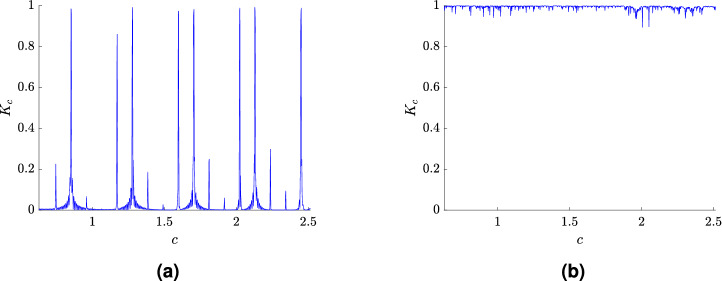
A plot of $K_c$ depending on $c$: (**a**) for $k_f = 3\times 10^{-4}$ showing regular dynamics, (**b**) for $k_f = 3.5\times 10^{-4}$ showing chaotic dynamics.

For these simulations, the free software environment R^33^ was used, including the *Chaos01* package developed by T. Martinovič^40^. A comparison of the values for $K_c$ for the periodic and chaotic cases is shown in Fig. 5, for $k_f = 3\times 10^{-4}$ and $k_f = 3.5\times 10^{-4}$, respectively.

**Figure 6:**
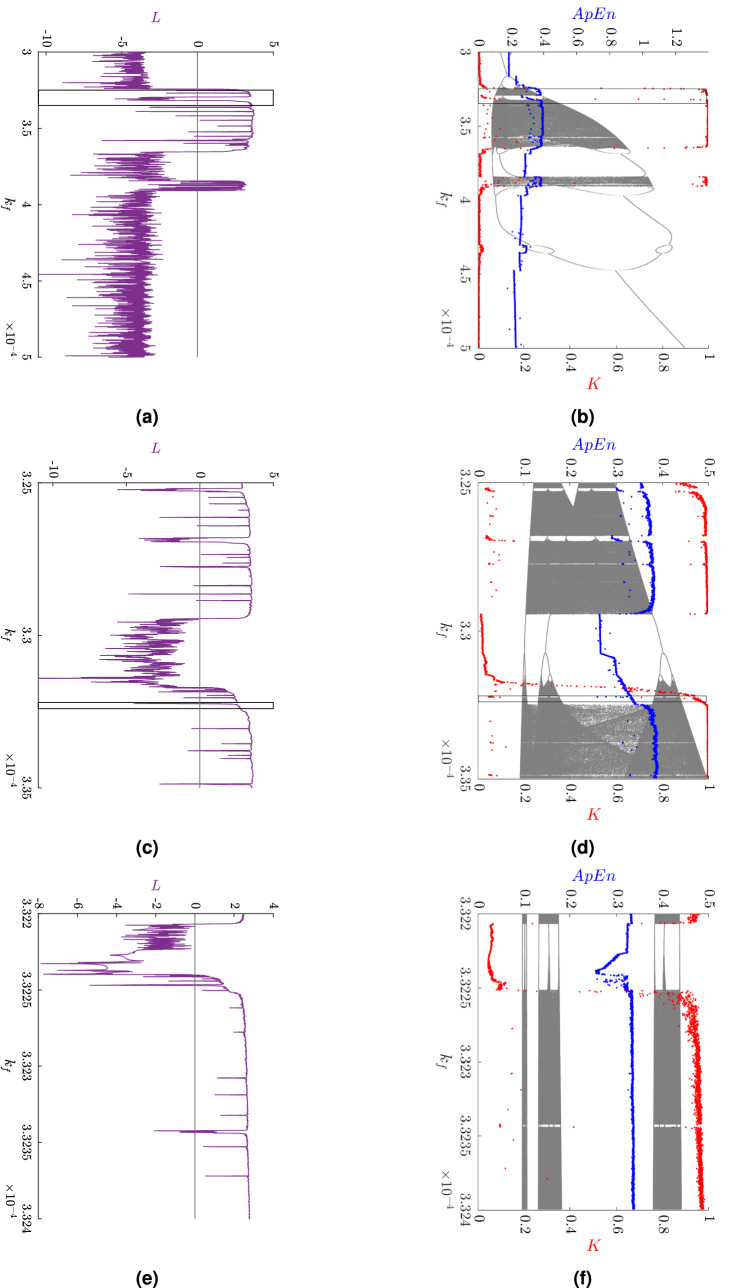
The dynamics characteristics of: (Left) the maximal Lyapunov exponent *L* [(**ac**,**e**), in purple]; (Right) approximate entropy *ApEn* [(**b**,**d**,**f**), in blue], and the result of the 0–1 test for chaos *K* [(**b**,**d**,**f**), in red]; the bifurcation diagram for variable *x* is shown in the background. The magnification of both sub-intervals denoted by the black rectangle is shown on the figure: (**a**,**b**) the results for $k_f \in (3\times 10^{-4},5\times 10^{-4})$, (**c**,**d**) the results for $k_f \in (3.25\times 10^{-4},3.35\times 10^{-4})$, (**e**,**f**) the results for $k_f \in (3.322\times 10^{-4},3.324\times 10^{-4})$.

The results of the 0–1 test for chaos for all values of the parameter $k_f$ are shown in Fig. 6.

## Discussion

In this paper the detection of embedded dynamics of the GF model (2), associated with the BZ chemical reaction, was intensively researched. For this purpose, the GF model was solved using the adaptive six-stage, fifth-order, Runge–Kutta method implemented as the $ode45$ solver in Matlab. To eliminate the stiffness problem, the model (2) was also simulated by the $ode23s$ solver in Matlab^28^; the outputs were identical.

The simulations were used to plot 3D phase portraits, bifurcation diagrams, the approximate entropy, the 0–1 test for chaos, and the maximal Lyapunov exponent. The search process of dynamical properties, depending on the flow rate parameter $k_f$, was performed in the free software R^33^ using the packages $TSEntopies$^32^ and $Chaos01$^40^, and the Lyapunov exponent calculation Matlab script^41^. It is also wort noting that there are other possibilities for detecting dynamic properties (e.g. fractal dimension), see e.g.^42,43^.

It is evident from the main results shown in Fig. 6 that all tests clearly detect regular and irregular patterns for a given $k_f$.

Firstly, bifurcation analysis detects regions of flow rate parameters where only cycle trajectories appear, and also those values of $k_f$ where chaos is assumed. Nevertheless, typical properties of bifurcation analysis were observed. Moreover, the reverse bifurcation phenomenon is well visible, that is if $k_f$ stands for chaos and increases, the system’s complexity decreases, reaching cycle trajectories. Our results show that the method of approximate entropy returns a qualification constant which describes complexity in the system invariantly with respect to the origin. On the other hand, the 0–1 test as a qualification tool returns zero for regular (periodic or quasi-periodic) trajectory, and one for irregular (chaotic) characteristics.

Moreover, if the output of the 0–1 test is not close to zero or one, then the examined test case has not yet reached the attractor or has reached an intermittent state, see e.g.^44,45^ and references therein. These results are underlined by the maximal Lyapunov exponent, which detects not only periodic and chaotic trajectories, but also bifurcation borders. As is observable from Fig. 6, the outputs of all these tests are well associated.

Further, we observe a correlation between the approximate entropy and the 0–1 test for chaos. In general, the increasing values of the 0–1 test for chaos are coupled to increasing approximate entropy and vice versa.

We notice isolated low values of the 0–1 test for chaos accompanied by comparatively low values of approximate entropy well within the chaotic region characterized by high 0–1 test for chaos values and approximate entropy. To investigate and zoom in, we constructed a three-stage system of nested sub-intervals of flow rates $k_f$, see Fig. 6, for which in every level the 0–1 test for chaos and approximate entropy was computed. At every level we observed the same pattern. This naturally suggests a fractal structure in the set of $k_f$:

### **Open Problem 1**

Is there a totally disconnected (Cantor) set of flow rates $k_f$ in $[3\times 10^{-4}, 5\times 10^{-4}]$ such that for each such parameter the GF model (2) is showing chaos?

### **Open Problem 2**

Is there a totally disconnected (Cantor) set of flow rates $k_f$ in $[3\times 10^{-4}, 5\times 10^{-4}]$ such that for each such parameter the GF model (2) is showing regular pattern?
